# The making of a killer (T cell)

**DOI:** 10.18632/oncotarget.15229

**Published:** 2017-02-09

**Authors:** Willem W. Overwijk

**Affiliations:** Department of Melanoma Medical Oncology, University of Texas MD Anderson Cancer Center, Houston, TX, USA

**Keywords:** T cells, cancer, antigen, culture, adoptive transfer

Adoptive T cell therapy (ACT) is a powerful anti-cancer modality - if you can get the T cells. In an exciting new study, Pathangey *et al*. [1] diligently define how to grow large numbers of tumor antigen-specific “helper” and “killer” Tcells from peripheral blood, addressing a major bottleneck in bringing ACT to more patients with cancer.

Currently, there are four major ways to obtain tumor-specific T cells for ACT: 1) Perform a partially mismatched bone marrow transplant, leading to the induction of a graft *vs*. host T cell response often accompanied by a graft *vs.* tumor response; 2) Isolate tumor-infiltrating lymphocytes (TIL) from a surgically removed tumor and numerically expand these *ex vivo*; 3) Genetically engineer T cells from peripheral blood mononuclear cells (PBMC) to express either a tumor-specific T cell receptor (TCR) or chimeric antigen receptor (CAR) consisting of the variable domain of an antibody fused to the intracellular portion of the TCR, often with inclusion of additional positive signaling domains; or 4) Prime and expand tumor-specific T cells from PBMC (or cord blood cells) *ex vivo* with tumor antigen. Thus far, options 1 and 3 have worked well against some leukemias and lymphomas, but solid tumors appear much less responsive [2]. Option 2 works well for patients with melanoma, but in other cancers it is difficult to reproducibly grow TIL with good tumor recognition, let alone clinical efficacy [3]. Pathangey et al. combined intelligent reasoning and brute-force empiricism to design a novel method for the *in vitro* generation of tumor antigen-specific T cells from easy to obtain PBMC.

The new method is grounded in 2 major concepts: 1) T cells are activated by antigen-presenting cells (APC) such as dendritic cells (DC), which themselves require proper innate activation signals to function; and 2) Activated T cells need a cytokine signal to proliferate and survive [4]. To grow DC, the team cultured whole PBMC in a classic media with GM-CSF (GM) and IL-4, then added combinations of the known DC activators poly I:C, lipopolysaccharide (LPS), resiquimod (R848) and interferon (IFN)-γ. Based on DC yield and function, the best innate activation cocktail consisted of GM+R848+LPS. The next day, long peptides were added containing known and newly predicted, HLA-binding T cell epitopes from the MUC1 and Her2 antigenic proteins, widely expressed in many cancers. Experiments with inhibitors of peptide processing revealed that the long peptides were ingested by DCs, trimmed and bound to HLA molecules, then presented on the DC surface to the T cells to induce their activation.

The next step involved the testing of combinations of 4 cytokines known to signal through the common-γ chain cytokine receptor subunit to promote T cell proliferation and survival. Through the years, different groups have used the common-γ chain agonist cytokines IL-2, IL-7, IL-15, IL-21 and combinations thereof to expand DC-primed T cells. Surprisingly, Pathangey *et al*. found that among an extensive series of variously timed exposures to different cytokine combinations a simple culture with IL-7 alone, or with IL-7 and IL-2, yielded the largest number of antigen-specific CD4+ and CD8+ T cells. While IL-2 and IL-7 were each able to induce proliferation, only IL-7 supported long-term survival, yielding the greatest number of antigen-specific T cells after 19 days. These T cells were functional, killing and producing cytokines upon contact with antigen-positive tumor cells. The team was able to generate CD4+ and CD8+ T cells with specificity for multiple cancer and virus-related antigenic epitopes, from multiple healthy donors and cancer patients, indicating the robustness of the methodology.

A critical lesson from this study is that the particular combination of innate stimuli determined the success of the *ex vivo* T cell generation. Adding R848 and LPS to the cultures predictably increased DC expression of costimulatory molecules. More surprisingly, numeric expansion of the cultures was not altered by addition of R848 and/or LPS, but the fraction of antigen-specific cells was dependent on the exact combination of innate stimuli. This raises questions about the exact molecules, costimulatory or otherwise, that primed the T cells for subsequent optimal expansion with IL-7. Similarly, while the authors found no specific impact of IL-7 on the expression of the pro-survival molecule, Bcl-2, IL-7R signaling also drives expression of the bcl-2 family member Mcl-1, which is a major mediator of T cell survival by inhibiting Bak, and could explain IL-7's remarkable ability to promote T cell survival [4, 5].

T cell survival, indeed, is what the present study begs to be investigated next. While the ability to reproducibly raise tumor-specific T cells is critical for the implementation of ACT in solid tumors beyond melanoma, it is now clear that both the antigen-sensitivity and the *in vivo* proliferation and persistence of these T cells directly determine clinical efficacy [6, 7]. Thus a critical next step is to measure the ability of the *ex vivo* generated T cells to recognize tumor cells endogenously expressing different amounts of antigen and HLA. Perhaps even more importantly, will these T cells survive in the patient? Pre-conditioning, non-myeloablative chemotherapy regimens can raise endogenous levels of IL-7, as well as IL-15, *in vivo* [8], and IL-2 is routinely administered in ACT therapy with TIL. IL-7 and IL-15 have been safely administered in clinical trials, and could support *ex vivo* generated T cells for ACT [4]. Some of this work could conceivably be done in immunodeficient animals bearing human tumors, and supplied with the T cells and human cytokines, but the true test will be in patients with cancer. It will be exciting to follow Pathangey *et al*.'s work as it makes its way from the lab to the clinic. May it prove a key to making the power of ACT more widely available to patients with cancer.
